# Blockade of CC Chemokine Receptor Type 3 Diminishes Pain and Enhances Opioid Analgesic Potency in a Model of Neuropathic Pain

**DOI:** 10.3389/fimmu.2021.781310

**Published:** 2021-11-02

**Authors:** Katarzyna Pawlik, Agata Ciechanowska, Katarzyna Ciapała, Ewelina Rojewska, Wioletta Makuch, Joanna Mika

**Affiliations:** Department of Pain Pharmacology, Maj Institute of Pharmacology, Polish Academy of Sciences, Krakow, Poland

**Keywords:** CCR3, neutrophils, morphine, buprenorphine, cytokines, 4-aminobenzoic hydrazide, myeloperoxidase, SB328437

## Abstract

Neuropathic pain is a serious clinical issue, and its treatment remains a challenge in contemporary medicine. Thus, dynamic development in the area of animal and clinical studies has been observed. The mechanisms of neuropathic pain are still not fully understood; therefore, studies investigating these mechanisms are extremely important. However, much evidence indicates that changes in the activation and infiltration of immune cells cause the release of pronociceptive cytokines and contribute to neuropathic pain development and maintenance. Moreover, these changes are associated with low efficacy of opioids used to treat neuropathy. To date, the role of CC chemokine receptor type 3 (CCR3) in nociception has not been studied. Similarly, little is known about its endogenous ligands (C-C motif ligand; CCL), namely, CCL5, CCL7, CCL11, CCL24, CCL26, and CCL28. Our research showed that the development of hypersensitivity in rats following chronic constriction injury (CCI) of the sciatic nerve is associated with upregulation of CCL7 and CCL11 in the spinal cord and dorsal root ganglia (DRG). Moreover, our results provide the first evidence that single and repeated intrathecal administration of the CCR3 antagonist SB328437 diminishes mechanical and thermal hypersensitivity. Additionally, repeated administration enhances the analgesic properties of morphine and buprenorphine following nerve injury. Simultaneously, the injection of SB328437 reduces the protein levels of some pronociceptive cytokines, such as IL-6, CCL7, and CCL11, in parallel with a reduction in the activation and influx of GFAP-, CD4- and MPO-positive cells in the spinal cord and/or DRG. Moreover, we have shown for the first time that an inhibitor of myeloperoxidase-4-aminobenzoic hydrazide may relieve pain and simultaneously enhance morphine and buprenorphine efficacy. The obtained results indicate the important role of CCR3 and its modulation in neuropathic pain treatment and suggest that it represents an interesting target for future investigations.

## Introduction

According to the International Association for the Study of Pain (IASP), neuropathic pain is defined as ‘pain caused by a lesion or disease of the somatosensory nervous system’. This chronic pathology is divided into central or peripheral neuropathy (www.iasp-pain.org). Despite many years of research, the mechanism underlying this phenomenon is not fully understood, resulting in ineffective pain therapies and treatment, as even strong analgesics such as opioids appear to be inefficient (1, 2). Recent evidence suggests that immune factors and their interactions are involved in the development and persistence of neuropathy (3–5). Among them, chemokines appear to play important roles in the pathogenesis of neuropathic pain (6–9). These small cytokines and their receptors are produced and expressed by neuronal and nonneuronal cells. Some of those cells (e.g. neurons, microglia, astrocytes, and lymphocytes) have been confirmed to be involved in the development and maintenance of neuropathic pain (10–17). Previous studies, including studies from our group, indicate that the intrathecal administration of chemokines such as CCL2, CCL3, CCL4, CCL7 and CCL9 may evoke hypersensitivity to mechanical and thermal stimuli (18–20). Moreover, neutralization of chemokines (e.g., CCL2, CCL3, CCL4, CCL7, and CXCL1) may reduce hypersensitivity and the inflammatory response (18, 21–26) in animal models of neuropathy. Interestingly, blockade of some chemokine receptors by antagonists (e.g. CCR1, CCR2, CCR5, CXCR3, and CX3CR1) diminishes pain-like behaviors and/or enhances opioid effectiveness in parallel with the modulation of immune factors after nerve injury in rats (27–32). Therefore, neuroimmune interactions, including chemokine signaling, seem to be crucial in the pathogenesis of neuropathic pain.

To our knowledge, no information is available on the contribution of C-C chemokine receptor 3 (CCR3) to the pathogenesis of neuropathic pain. The expression of CCR3 has been confirmed in cells modulating neuropathic pain development, including neurons, microglia, astrocytes, neutrophils and T cells (33–38). Interestingly, researchers suggest an important role for CCR3 in disorders such as asthma, cancer, atopic skin inflammation, narcolepsy and inflammatory bone resorption (39–43), but no information is available showing the involvement of the abovementioned receptor in pathological nociception. CCR3 has a few endogenous ligands: CCL5, CCL7, CCL11, CCL24, CCL26, and CCL28. Three of them, CCL5, CCL7, and CCL11, appear to be important factors involved in nociception processes (6, 18, 44–47).

The aim of the present study was to assess the changes in the expression of all of these ligands after chronic constriction injury (CCI) in a rat model of neuropathic pain by measuring their mRNA levels in two different structures of the nervous system, the spinal cord and dorsal root ganglia (DRG), at different time points after surgery. Moreover, we determined how the blockade of CCR3 by its potent and selective antagonist SB328437 affected pain-related behavior in CCI-exposed rats and, at the same time, how this blockade altered the protein levels of markers of microglia/macrophages (ionized calcium binding adapter molecule 1; IBA-1), astrocytes/satellite cells (glial fibrillary acidic protein; GFAP), T cells (CD4 and CD8), neutrophils (myeloperoxidase; MPO) and factors with pronociceptive (IL-1beta, IL-18, and IL-6) and antinociceptive (IL-10, IL-1RA, and IL-1BP) properties released by those cells. Additionally, we analyzed whether the coadministration of SB328437 with morphine or buprenorphine increased their analgesic properties. Moreover, based on the obtained results, we studied whether coadministration of myeloperoxidase inhibitor with morphine or buprenorphine improved opioid effectiveness.

## Materials and Methods

### Animals

Male Wistar rats (275–300 g, Charles River, Hamburg, Germany) and Albino Swiss mice (20–25 g, Charles River) were used in our study. Rodents were housed in cages with sawdust bedding on a standard 12 h/12 h light/dark cycle (lights on at 06.00 a.m.), with food and water available *ad libitum*. Rats and mice were separated from each other. Experiments were carried out according to the recommendations and standards of the International Association for the Study of Pain (IASP) and the National Institutes of Health (NIH) Guide for the Care and Use of Laboratory Animals and were approved by the Ethical Committee of the Maj Institute of Pharmacology of the Polish Academy of Sciences (LKE: 116/2021; 213/2021; 1277/2015). According to the 3R policy, the number of animals was reduced to the necessary minimum.

### Catheter Implantation

Rats were implanted with catheters for intrathecal (*i.t.*) injections according to method reported by Yaksh and Rudy (48). Before surgery, animals were anesthetized with sodium pentobarbital (60 mg/kg) *via* intraperitoneal (*i.p.*) injection. Thirteen cm-long polyethylene catheters (PE 10, Intramedic; Clay Adams, Parsippany, USA) with a dead space of 10 μl were sterilized by immersion in 70% (v/v) ethanol and catheter was rinsed thoroughly with water for injection, immediately before surgery. During insertion, 7.8 cm of each catheter was introduced through the atlanto-occipital membrane into the subarachnoid space at the rostral level of the spinal cord lumbar enlargement (L4-L5). After the process, 10 μl of water were injected, and the catheters were tightened. The rats recovered for 1 week before the next surgery. The surgery was performed under aseptic conditions. No catheters were implanted in mice.

### Neuropathic Pain Model

Chronic constriction injury of the sciatic nerve in rats was performed under sodium pentobarbital anesthesia (60 mg/kg, *i.p*.), while in mice under isoflurane anesthesia. According to the Bennet and Xie (49) method and our previous studies (27, 29), the animals under anesthesia had their skin cut at the height of the hip joint towards the knee joint. The lateral head of the *musculus vastus lateralis* was then separated from the *musculus biceps femoris*, which allowed access to the sciatic nerve under *the musculus gluteus maximus*. Gently using tweezers, passing between the *musculus semimembranosus* and the sciatic nerve. Exposed sciatic nerve was tied, four times in case of rats and three times in case of mice, using ligatures (4/0 silk) with 1-mm intervals, until a brief twitch (when placing first ligature) in the operated hind limb was observed. Next the skin was sutured. The surgery was performed under aseptic conditions.

### Drug Administration

#### Single Intrathecal Administration of Drugs in Rats

In this experiment, we used the CCR3 antagonist SB328437 [SB, N-(1-naphthalenylcarbonyl)-4-nitro-L-phenylalanine methyl ester, (Tocris, Bristol, UK)] dissolved in 70% dimethyl sulfoxide (V; DMSO). A group of rats received a single injection of SB328437 (SB; 10 μg/5 μl, *i.t.*) or vehicle (V, dissolved in water for injection) on the 12^th^ day post-CCI, when fully developed mechanical and thermal hypersensitivity was observed, and behavioral reactions were measured using von Frey and cold plate tests, respectively. Additionally, naive rats were used in the experiment.

#### Repeated Intrathecal Administration of Drugs in Rats

In the experiment, we used the following substances: SB328437 (SB), morphine hydrochloride (M; Fagron, Krakow, Poland), and buprenorphine (B; Polfa Warszawa S+A, Warsaw, Poland). SB328437 was dissolved in vehicle (V), whereas morphine and buprenorphine were dissolved in water for injection.

The first group of rats received a pretreatment with 10 μg/5 μl *i.t*. SB 16 and 1 h before CCI surgery and then once a day for the following 7 days. The control rats were administered vehicle (V) according to the same schedule. Behavioral tests (von Frey and cold plate tests) were performed on the 2^nd^ and 7^th^ days post-CCI 3 h after the vehicle or SB injection (**Figure 4A**). Additionally, naive rats were used in the experiment.

Moreover, on the 6^th^ day after CCI surgery, other group of the chronically treated rats received a single dose of vehicle (V_;_ water for injection) morphine (2.5 μg/5 μl, *i.t.*) or buprenorphine (1.0 μg/5 μl*, i.t.*) 30 min after V (70% DMSO) or CCR3 antagonist administration. These animals performed the same behavioral tests 30–35 min after V or opioid injection (it is 1 h after V or SB injections) (**Figure 5A**).

#### Single Intraperitoneal Administration of Drugs in Mice

In the experiment, we used the following substances: myeloperoxidase inhibitor, 4-aminobenzoic hydrazide ( 4A; Sigma–Aldrich, St. Louis, USA), morphine (M), buprenorphine (B) and SB328437 (SB). 4A, M and B were dissolved in water for injection, while SB were dissolved in 70% DMSO. First group of mice received vehicle (V; water for injection) or 4-aminobenzoic hydrazide (60 mg/kg, *i.p.*), 6 days after surgery and then 30 min after V or SB delivery, V, morphine (5 mg/kg, *i.p.*) and buprenorphine (5 mg/kg, *i.p.*) were injected. 30 to 35 min after the vehicle or opioid injection (it is 1 h after V or 4A injections), von Frey and cold plate tests were performed (**Figure 10A**). Another group of animals received single dose of vehicle (70% DMSO) or SB (20 μg/5 μl*, i.t.*) on the day 2^nd^ after CCI. Behavioral tests were performed 1 h, 5h and 24 h after substances administration. Additionally, naive mice were used in the experiment.

### Behavioral Tests

#### Mechanical Hypersensitivity Measurement (von Frey Test)

A von Frey apparatus with a strength up to 26 g (Dynamic Plantar Anesthesiometer, Ugo Basile, Gemonio, Italy) was used to measure mechanical hypersensitivity in rats, while calibrated nylon monofilaments with 0.6–6 g strength (Stoelting, Wood Dale, USA) were used to measure hypersensitivity in mice as previously described (32, 50). 5 minutes before the measurements, the animals were placed in plastic cages with a wire-net floor and were not restricted in their mobility. The touch stimulator of the machine or filaments were moved under the operated hind limb, and the reaction of the animal to the stimulus was measured automatically (for rats) or until the animal lifted the injured paw (for mice). The reaction of both hind paws in naive rodents was measured.

#### Thermal Hypersensitivity Measurement (Cold Plate Test)

A cold plate analgesia meter (Columbus Instruments, Columbus, USA and Ugo Basile, Gemonio, Italy) was used to measure thermal hypersensitivity in rats and mice, respectively, as previously described (27, 29). The rodents were placed on the cold surface (5°C for rats; 2°C for mice) and remained there until they lifted the operated hind paw. The cutoff latency for both species was 30 s. Reactions of both hind paws were observed in naive rodents.

### Analysis of Gene Expression

#### Reverse Transcription Quantitative Real-Time PCR (RT–qPCR)

Dorsal lumbar segments of the spinal cord (L4–L6) and the DRG (L4–L6) were collected from CCI-exposed rats after decapitation on the 2^nd^, 7^th^, 14^th^ and 28^th^ days post‐CCI and from naive animals immediately after behavioral test performance. According to Chomczynski and Sacchi (51), total RNA was extracted using TRIzol reagent (Invitrogen, Carlsbad, USA). The concentration and quality of RNA were measured using a DeNovix DS-11 spectrophotometer (DeNovix Inc., Wilmington, USA). The Omniscript RT Kit (Qiagen Inc., Hilden, Germany), oligo (dT16) primer (Qiagen Inc., Hilden, Germany), and RNAse inhibitor (rRNasin, Promega, Mannheim, Germany) were used to perform reverse transcription of 1 μg of total RNA at 37°C. The obtained cDNA templates were diluted 1:10 using RNase-/DNase-free H_2_O. RT–qPCR was conducted with approximately 50 ng of cDNA templates from each sample using Assay-On-Demand TaqMan probes (Applied Biosystems, Foster City, USA) and an iCycler device (Bio-Rad, Hercules, Warsaw, Poland). The following TaqMan primers were used: *CCL5* (Rn00579590_m1), *CCL7* (Rn01467286_m1), *CCL11* (Rn00569955_m1), *CCL24* (Rn01481451_m1), *CCL26* (Rn01481484_m1*), CCL28* (Rn00586715_m1), *CCR3* (Rn02134292_s1), and *Hprt* (Rn01527840_m1). *Hprt* was used as an endogenous control and an adequate housekeeping gene since it did not exhibit any significant changes among groups. The cycle threshold values were automatically calculated using CFX Manager v.2.1 software with the default parameters. The RNA content was calculated using the formula 2^−(threshold cycle)^.

### Analysis of Protein Levels

#### Western Blotting

Tissues from ipsilateral spinal cord segments (L4-L6) and DRGs were collected 6 hours after the last V or SB328437 administration on day 7 post-CCI and from rats after sciatic nerve surgery in the absence of drug injections on days 2, 7, 14 and 28. Next, samples were placed in RIPA buffer supplemented with a protease inhibitor cocktail (Sigma–Aldrich, St. Louis, USA), homogenized and cleared *via* centrifugation (30 min, 14000 rpm, 4°C). The total protein concentration was measured using the bicinchoninic acid method. The obtained samples (10 µg of protein) were heated in a mix of loading buffer (4x Laemmli Buffer, Bio-Rad, Warsaw, Poland) containing 2-mercaptoethanol (Bio-Rad, Warsaw, Poland) for 8 min at 98°C. Electrophoresis was performed using 4–15% Criterion™ TGX™ precast polyacrylamide gels (Bio-Rad, Warsaw, Poland). In the next step, the proteins were transferred (semidry transfer 30 min, 25 V) to Immune-Blot PVDF membranes (Bio-Rad, Warsaw, Poland) and then blocked for 1 h at RT using 5% nonfat, dry milk (Bio-Rad, Warsaw, Poland) in Tris-buffered saline containing 0.1% Tween-20 (TBST). After transfer, the membranes were washed with TBST buffer and incubated overnight (4°C) with the following primary antibodies: rabbit: anti-CCR3 (1:1000; Novus, Abingdon, UK), anti-IL-1beta (1:500, Abcam, Cambridge, UK), anti-IL-6 (1:500, Invitrogen, Carlsbad, CA), anti-IL-18 (1:1000, Abcam, Cambridge, UK), anti-IL-18BP (1:500, Novus, Abingdon, UK), anti-IL-10 (1:1000, Abcam, Cambridge, UK), anti-IL-1RA (1:2000, Abcam, Cambridge, UK), anti-IBA-1 (1:500; Novus, Abingdon, UK), anti-GFAP (1:10 000; Novus, Abingdon, UK), anti-CD8 (1:500; Santa Cruz, Dallas, TX), and anti-MPO (1:1000; Abcam, Cambridge); mouse: anti-CD4 (1:1000; R&D Systems, Minneapolis, USA) and anti-GAPDH (1:5000, Millipore, Darmstadt, Germany). The next day, the membranes were washed with TBST buffer and incubated for 1 h at RT with HRP-conjugated anti-rabbit or anti-mouse secondary antibodies (1:5000, Vector Laboratories, Burlingame, USA). SignalBoost™ Immunoreaction Enhancer Kit (Merck Millipore Darmstadt, Germany) was used to dilute primary and secondary antibodies. Proteins were detected using Clarity™ Western ECL Substrate (Bio-Rad, Warsaw, Poland) and visualized using the Fujifilm LAS-4000 FluorImager system. Fujifilm MULTI GAUGE software was used to estimate the levels of immunoreactive bands.

#### Enzyme-Linked Immunosorbent Assay

The samples were prepared for measurements as described above. ELISAs for CCL7 and CCL11 were performed according to the manufacturer’s instructions (rat C-C motif chemokine 7 ELISA Kit, ABclonal, Woburn, USA; Rat Eotaxin ELISA Kit, ABclonal, Woburn, USA). The detection limits for CCL7 and CCL11 were 15.6–1,000 pg/ml. Positive controls for the assays were provided by the manufacturer.

### Statistical Analysis

Data from behavioral studies are presented as the mean grams or seconds ± SEM. The intergroup differences were analyzed using one-way analysis of variance (ANOVA) followed by Bonferroni’s *post hoc* test for multiple comparisons. In the case of biochemical analyses, the results of RT–qPCR, Western blot and ELISA are presented as the fold change relative to the control (naive) ± SEM. Similar to the behavioral studies, the data were analyzed using one-way analysis of variance (ANOVA) followed by Bonferroni’s *post hoc* test. All data were analyzed using GraphPad Prism 8 software (GraphPad, San Diego, USA).

## Results

### Time Course of Changes in the mRNA Levels of CCL5, CCL7, CCL11, CCL24, CCL26 and CCL28 in the Spinal Cord and DRG and Related Pain Behaviors Measured on the 2^nd^, 7^th^, 14^th^ and 28^th^ Days After Chronic Constriction Injury of the Sciatic Nerve in Rats

**Figure 1:**
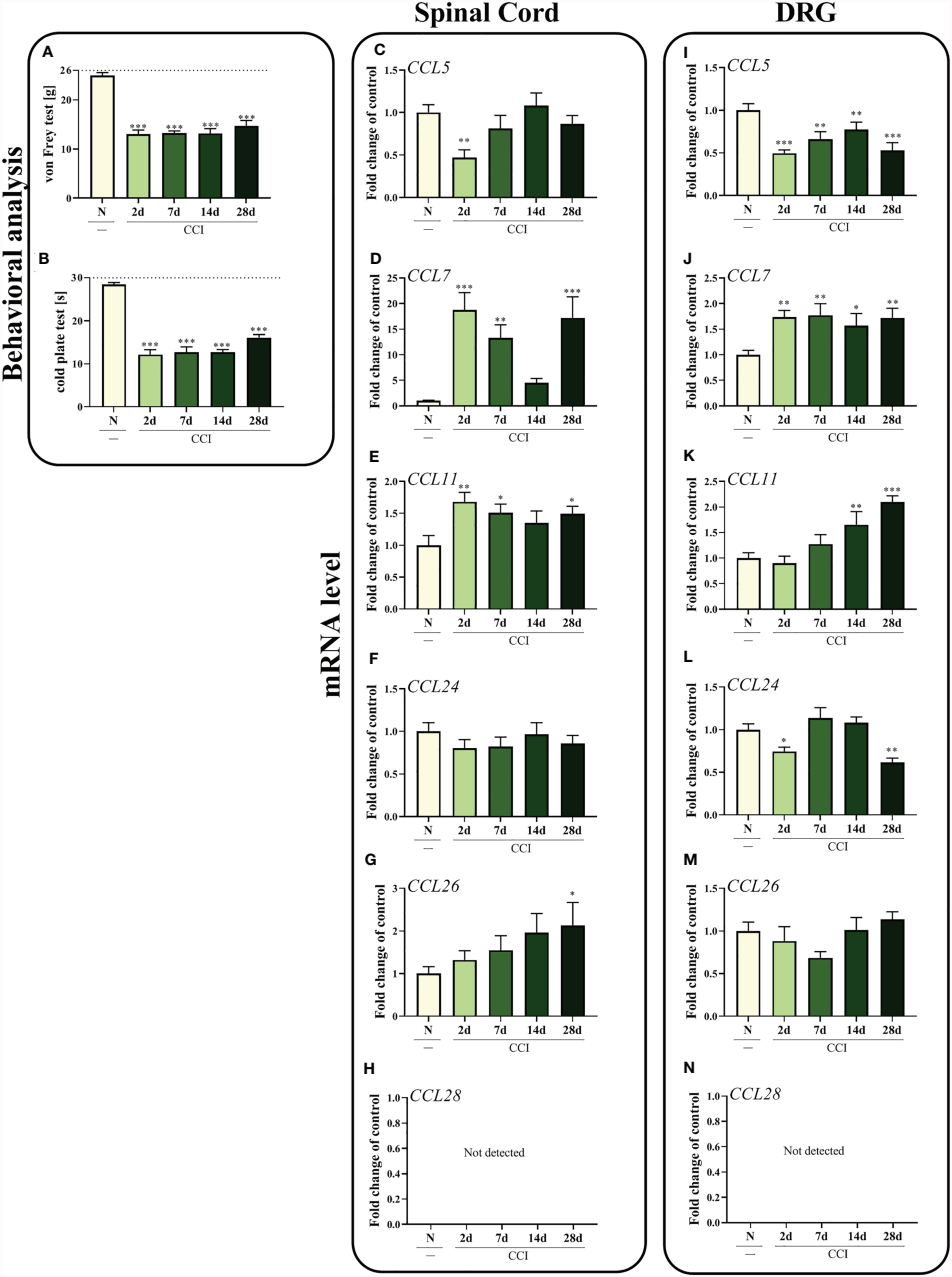
Time-dependent changes in pain-related behaviors in rats on the 2^nd^, 7^th^, 14^th^ and 28^th^ days after chronic constriction injury of the sciatic nerve [CCI; **(A)**, von Frey test; **(B)**, cold plate test] and associated changes in *CCL5, CCL7, CCL11, CCL24, CCL26*, and *CCL28* mRNA levels in the spinal cord **(C–H)** and DRG **(I–N)**. The behavioral data are presented as the means ± SEM of 10 rats per group. For behavioral studies **(A, B)**, the horizontal dotted line shows the cutoff value. The RT–qPCR data are presented as the means ± SEM of 6–9 samples per group. Intergroup differences were analyzed using ANOVA with Bonferroni’s *post hoc* test for multiple comparisons. *P < 0.05, **P < 0.01, and ***P < 0.001 indicate a significant difference compared with the control group (naive, N rats).

Sciatic nerve injury led to the development of mechanical (p < 0.0001; **Figure 1A**) and thermal (p < 0.0001; **Figure 1B**) hypersensitivity. These pain-related changes were observed until the last time point tested, as measured using both the von Frey test and cold plate test.

In the spinal cord, *CCL7* (p = 0.0001; **Figure 1D**) and *CCL11* (p = 0.0319; **Figure 1E**) seem to be important in the development of neuropathic pain, since increased levels of these mRNAs were observed 2 days after CCI. Interestingly, according to Bonferroni’s *post hoc* test, mRNA levels of those two chemokines together with *CCL26* (**Figure 1G**) were upregulated on day 28 after injury, which may prove their important roles in the maintenance of neuropathy. No changes in *CCL24* expression (**Figure 1F**) were observed in the spinal cord, while the level of *CCL5* (p = 0.0097; **Figure 1C**) was slightly downregulated 2 days after nerve ligation. As the only ligand of CCR3, *CCL28* (**Figure 1H**) was not detected in the spinal cord.

Similar results were obtained in the DRG to those in the spinal cord for *CCL7* (p = 0.0160; **Figure 1J**), where increased *CCL7* mRNA levels were observed in the early and late stages of neuropathic pain. The second upregulated chemokine was *CCL11* (p < 0.0001; **Figure 1K**); however, the changes started appearing 14 days after CCI and increased up to day 28. In contrast, significant downregulation of *CCL5* (p = 0.0005; **Figure 1I**) was observed at all time points examined, while a decrease in the *CCL24* mRNA level (p = 0.0001; **Figure 1L**) was observed on the 2^nd^ and 28^th^ days after nerve surgery. No changes in the expression of *CCL26* were detected (**Figure 1M**). As in the spinal cord, *CCL28* (**Figure 1N**) was not detected in the DRG.

### Time Course of Changes in the CCR3 mRNA and Protein Levels Measured in the Spinal Cord and DRG After Chronic Constriction Injury of the Sciatic Nerve in Rats

**Figure 2:**
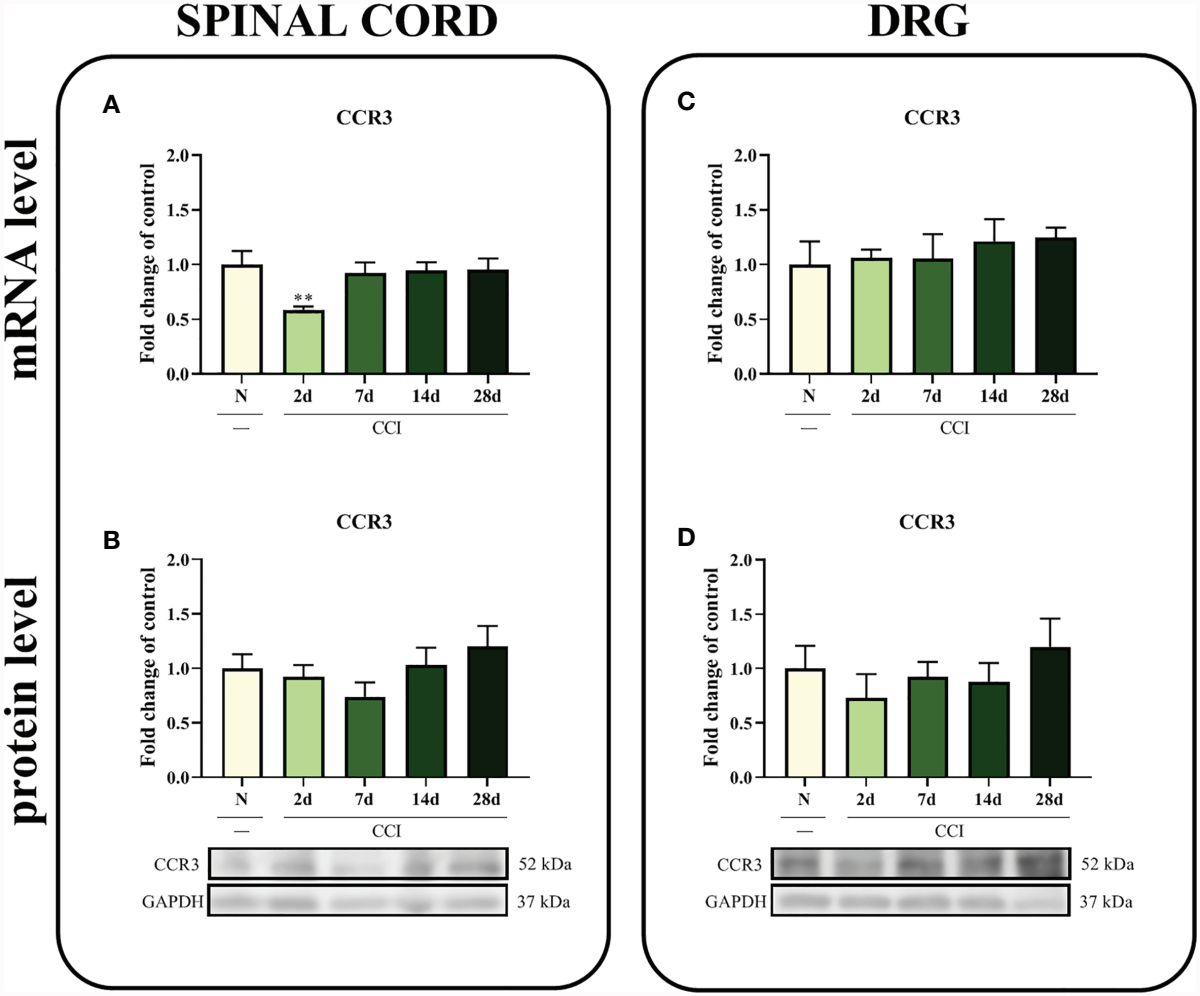
Time-dependent changes in the CCR3 mRNA and protein levels in the rat spinal cord **(A, B)** and DRG **(C, D)** on the 2^nd^, 7^th^, 14^th^ and 28^th^ days after chronic constriction injury of the sciatic nerve (CCI). The biochemical data are presented as the mean fold changes relative to the control ± SEM (4–9 samples per group). Intergroup differences were analyzed using ANOVA with Bonferroni’s *post hoc* test for multiple comparisons. **P < 0.01 indicates a significant difference compared with the control group (naive, N rats).

In the spinal cord, a significant difference in the level of the *CCR3* mRNA was observed 2 days after injury (p = 0.0208; **Figure 2A**); however, except for this time point, CCI did not alter the CCR3 mRNA and protein levels in the spinal cord (**Figures 2A, B** **)** or the DRG (**Figures 2C, D** **)**.

### Effects of Single and Repeated i.t. SB328437 Administration on Pain-Related Behaviors Measured 2, 7 and/or 12 Days After Chronic Constriction Injury in Rats and Mice

**Figure 3:**
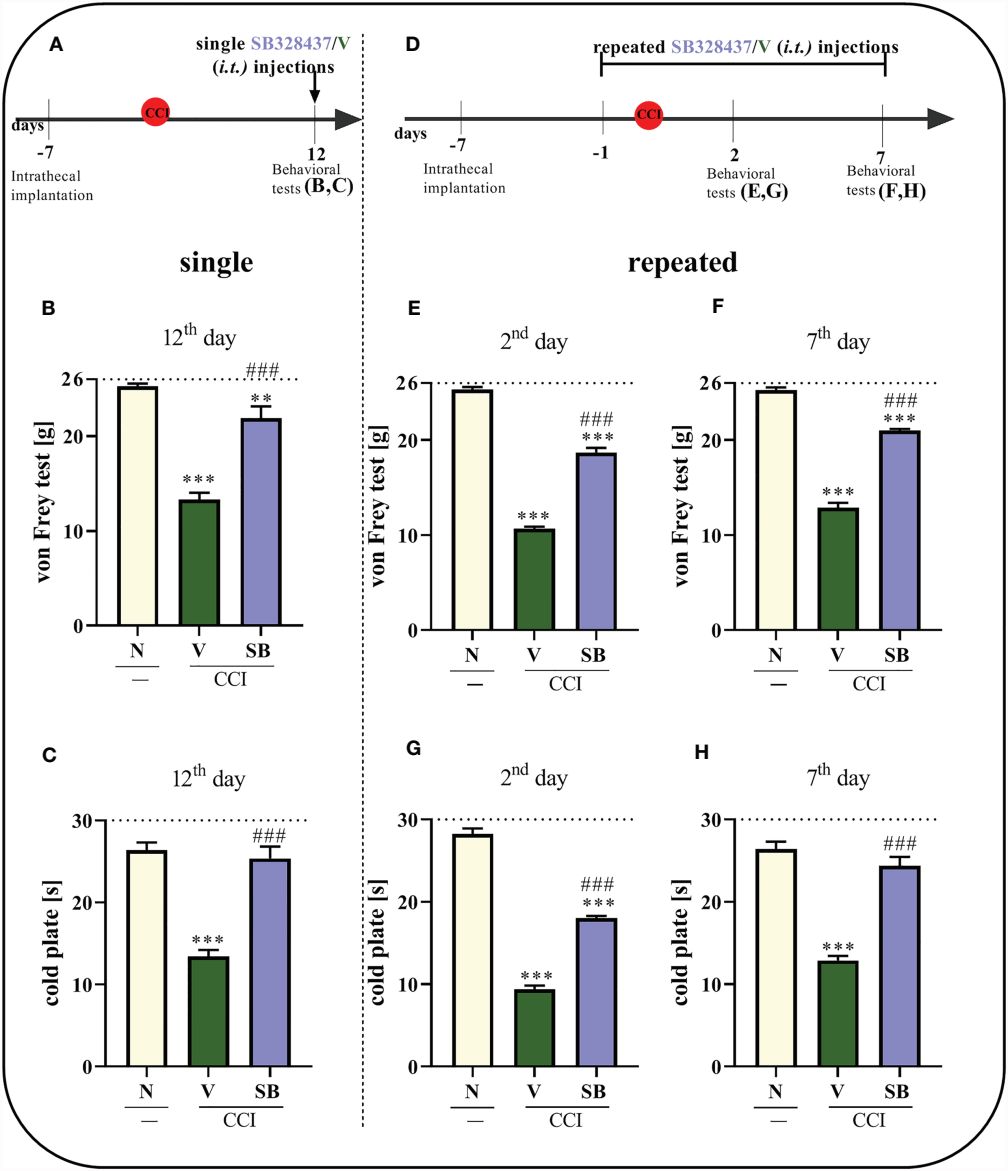
Effect of a single administration of SB328437 [SB, 10 μg/5 μl; *i.t*. **(A)**] on pain-related behaviors in rats on the 12^th^ day post- chronic constriction injury of the sciatic nerve (CCI), 3 h after drug injection [von Frey test- **(B)**; cold plate test- **(C)**] and repeated administration of SB in different group of rats, 16 h and 1 h before CCI and then once a day for 7 days on pain-related behaviors [10 μg/5 μl; *i.t*. **(D)**] as measured 2 [von Frey test- **(E)**; cold plate test- **(G)**] and 7 [von Frey test- **(F)**; cold plate test- **(H)**] days after chronic constriction injury. The horizontal dotted line shows the cutoff value. The data are presented as the means ± SEM (7–10 rats per group). Intergroup differences were analyzed using ANOVA with Bonferroni’s *post hoc* test for multiple comparisons. **P < 0.01 and ***P < 0.001 indicate a significant difference compared with the control group (naive, N rats); ^###^P < 0.001 indicates a significant difference compared with the Vehicle (V)-treated group.

Consistent with previously published literature, sciatic nerve surgery led to the development of mechanical and thermal hypersensitivity between days 2^nd^ and 12^th^, respectively, as measured using the von Frey test (p < 0.0001; **Figures 3B, E, F** **)** and cold plate test (p < 0.0001; **Figures 3C, G, H** **)**.

A single i.t. administration of the tested CCR3 antagonist (**Figures 3A**) reduced pain-related behaviors compared to vehicle treatment (**Figures 3B, C** **)** 12 days after nerve injury, so when the hypersensitivity was fully developed. The SB treatment prolonged the time the rats spent on the cold plate and increased the pain threshold for mechanical stimulation. Moreover, similar results we have obtained in CCI-exposed mice on the day 2^nd^ - the significant analgesic effect (p < 0.0001) was observed in both behavioral tests since 1 h till 5 h (von Frey: pretest- 0.96 g ± 0.1 *vs*. 1h- 2.81 g ± 0.2 *vs*. 5h- 1.93 g ± 0.1 *vs*. 24h- 0.98 g ± 0.1; cold plate: pretest- 13.05 s ± 0.7 *vs*. 1h- 18.91 s ± 0.5 *vs*. 5h- 17.04 s ± 0.6 *vs*. 24h- 12.15s ± 0.8).

In the case of repeated administrations (**Figure 3D**), the CCR3 antagonist diminished CCI-induced behavioral changes, as measured using the von Frey test (p < 0.0001; **Figure 3E**) and cold plate test (p < 0.0001; **Figure 3G**), compared to vehicle-treated animals after 2 days of daily administration. The same group of rats following 7 days of SB administration, exhibited even better analgesic properties, as measured using the cold plate test (p < 0.0001; **Figure 3H**), and effectively diminished mechanical hypersensitivity (p < 0.0001; **Figure 3F**).

### The Effect of Repeated i.t. Administration of SB328437 on Morphine and Buprenorphine Analgesic Effectiveness 6 Days After Chronic Constriction Injury of the Sciatic Nerve in Rats

**Figure 4:**
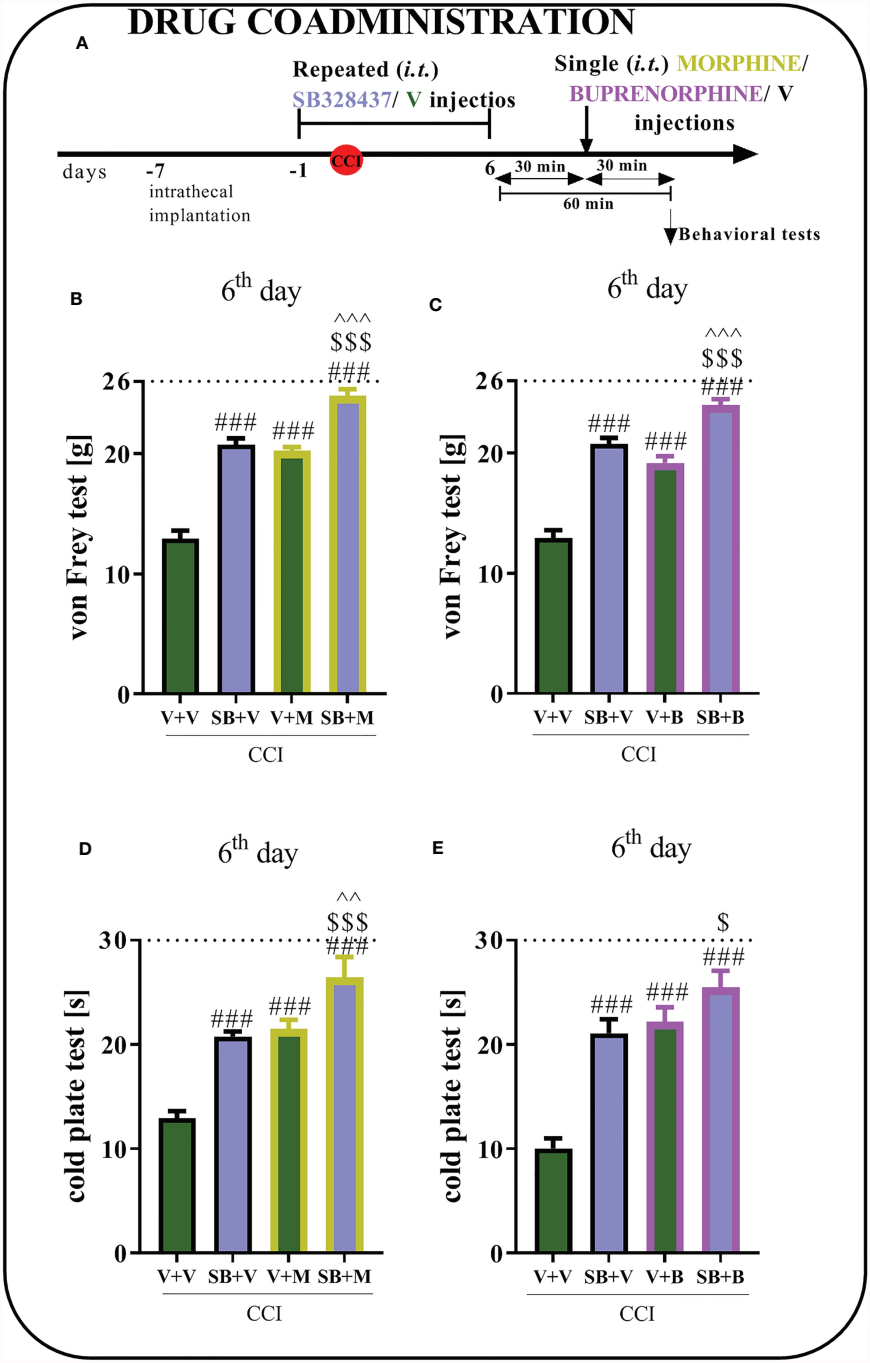
Effect of repeated administration of SB328437 [SB, 10 μg/5 μl; *i.t.* **(A)**] 16 h and 1 h before chronic constriction injury of the sciatic nerve (CCI) and then once a day for 6 days on pain-related behaviors in rats [von Frey test- **(B, C)** and cold plate test- **(D, E)**] and the analgesic effects of morphine (M) [2.5 μg/5 μl, *i.t*; **(B, D)**] and buprenorphine **(B)** [1.0 μg/5 μl, *i.t*. **(C, E)**] 1 h after SB or vehicle injections (30 min after opioids injections) on the 6^th^ day post-CCI. The data are presented as the means ± SEM of 5–7 rats per group. The horizontal dotted line shows the cutoff value. Intergroup differences were analyzed using ANOVA with Bonferroni’s *post hoc* test for multiple comparisons. ^###^P < 0.001 indicates a significant difference compared with the Vehicle (V)-treated group; ^$^P < 0.05 and ^$$$^P < 0.001 indicate a significant difference compared with the SB+V-treated group; ^^P < 0.01 and ^^^P < 0.001 indicate significant differences between the V+M- and SB+M-treated rats and between the V+B- and SB+B-treated rats.

Behavioral tests showed that repeated CCR3 antagonist administration for 6 days reduced mechanical and thermal hypersensitivity (**Figures 4B–E**), similar to the effect observed on day 7 (**Figures 4D, G** **)**. Moreover, the analgesic effects of morphine were stronger when morphine was coadministered with SB, as measured using von Frey (p < 0.0001; **Figure 4B**) and cold plate (p < 0.0001; **Figure 4D**) tests. When the antagonist was administered prior to buprenorphine, an increase in opioid efficacy was observed, as evidenced by the change in mechanical hypersensitivity (p < 0.0001; **Figure 4C**). Common administration of buprenorphine and SB revealed slightly reduced hypersensitivity in the cold plate test (**Figure 4E**) compared to administration of the opioid alone; however, the differences were not significant.

### Effect of Repeated i.t. SB328437 Administration on Protein Levels of Dactors With Pronociceptive Properties (IL-1beta, IL-6, and IL-18) in the Rat Spinal Cord and DRG 7 Days After Chronic Constriction Injury of the Sciatic Nerve in Rats

**Figure 5:**
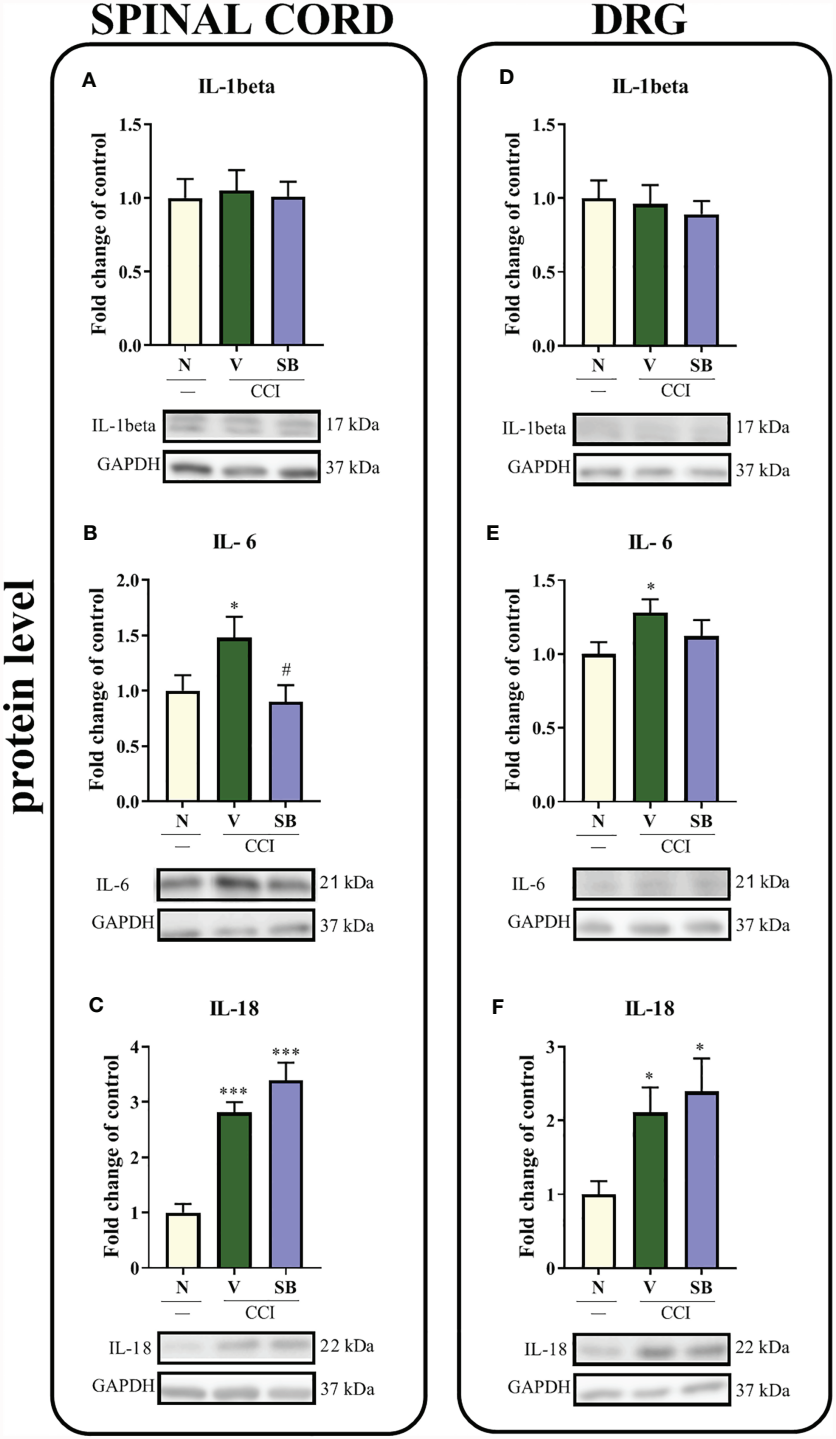
Effects of repeated administration of SB328437 (SB, 10 μg/5 μl; *i.t*.) 16 h and 1 h before CCI and then once a day for 7 days on the levels of the IL-1 beta **(A, D)**, IL-6 **(B, E)** and IL-18 **(C, F)** proteins in the spinal cord **(A–C)** and the DRG **(D–F)** on the 7^th^ day post- chronic constriction injury of the sciatic nerve (CCI) in rats. The data are presented as the mean fold changes relative to the control ± SEM (6–8 samples per group). Intergroup differences were analyzed using ANOVA with Bonferroni’s *post hoc* test for multiple comparisons. *P < 0.05 and ***P < 0.001 indicate a significant difference compared with the control group (naive, N rats); ^#^P < 0.05 indicates a significant difference compared with the Vehicle (V)-treated group.

No changes in IL-1beta protein levels were observed in either the spinal cord (**Figure 5A**) or DRG (**Figure 5D**) of vehicle- or SB-treated rats. In the spinal cord, chronic constriction injury increased the levels of the IL-6 (p = 0.0444; **Figure 5B**) and IL-18 proteins (p < 0.0001; **Figure 5C**). Additionally, significant downregulation of IL-6 was detected after CCR3 antagonist treatment compared to vehicle treatment. In the DRG, observations were similar to those in the spinal cord of vehicle-treated animals, where increased levels of IL-6 (**Figure 5E**) and IL-18 (p = 0.0239; **Figure 5F**) were detected. However, the tested substance was not able to prevent these changes in the levels of either protein.

### Effect of Repeated i.t. SB328437 Administration on the Protein Levels of Actors With Antinociceptive Properties (IL-10, IL-1RA, and IL-18BP) in the Rat Spinal Cord and DRG 7 Days After Chronic Constriction Injury of the Sciatic Nerve In Rats

**Figure 6:**
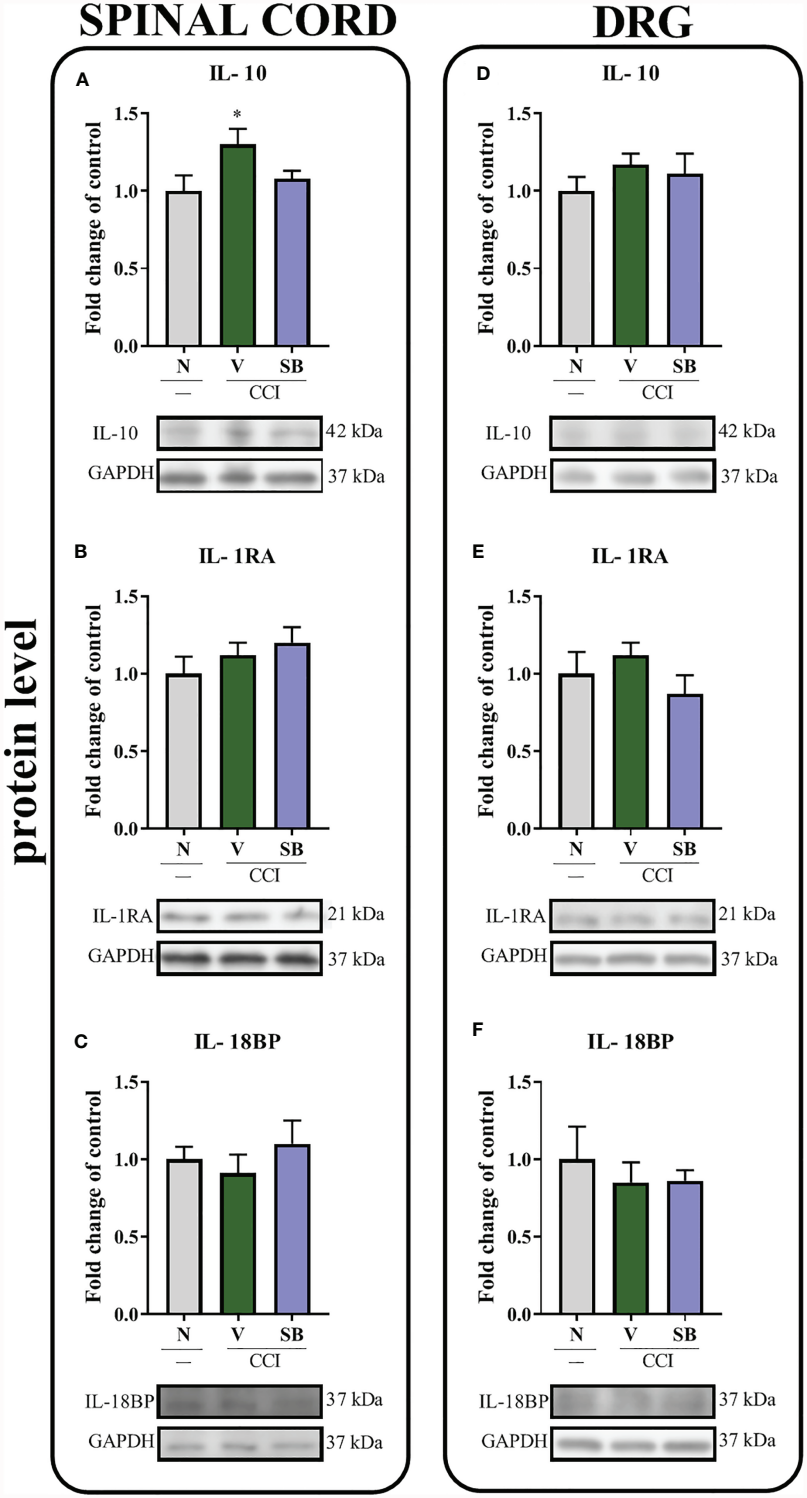
Effects of repeated administration of SB328437 (SB, 10 μg/5 μl; *i.t*.); 16 h and 1 h before chronic constriction injury of the sciatic nerve (CCI) and then once a day for 7 days on levels of the IL- 10 **(A, D)**, IL- 1RA **(B, E)** and IL-18BP **(C, F)** proteins in the rat spinal cord **(A–C)** and the DRG **(D–F)** on the 7^th^ day post-CCI. The data are presented as the mean fold changes relative to the control ± SEM (6–8 samples per group). Intergroup differences were analyzed using ANOVA with Bonferroni’s *post hoc* test for multiple comparisons. *P < 0.05 indicates a significant difference compared with the control group (naive, N rats). No differences were measured compared to Vehicle (V) group.

Sciatic nerve injury did not lead to changes in IL-1RA (**Figure 6B**) protein levels in the spinal cord. Interestingly, vehicle-treated, operated animals displayed increased levels of IL-10 (**Figure 6A**) in the same structure, according to Bonferroni’s *post hoc* test. However, repeated SB administration did not alter the levels of the aforementioned proteins compared to vehicle-treated rats. Moreover, no changes were observed in IL-18BP (**Figure 6C**) protein levels compared to naive animals. Additionally, neither nerve injury nor the CCR3 antagonist altered IL-10, IL-1RA and IL-18BP protein levels (**Figures 6D–F**) in the DRG.

### Effect of Repeated i.t. SB328437 Administration on Levels of the CCL7, CCL11 and CCR3 Proteins in the Rat Spinal Cord and DRG 7 Days After Chronic Constriction Injury of the Sciatic Nerve in Rats

**Figure 7:**
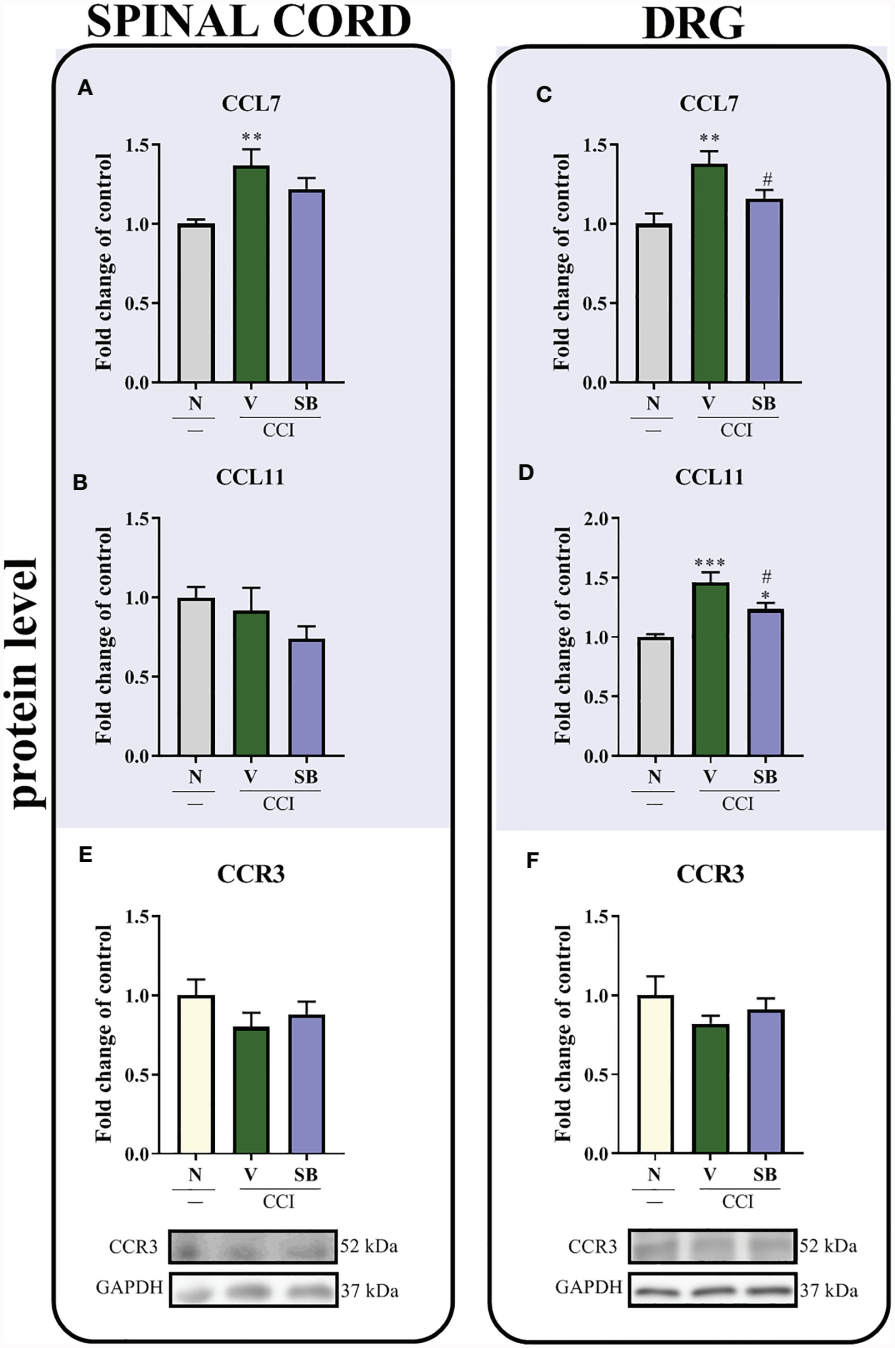
Effects of repeated administration of SB328437 (SB, 10 μg/5 μl; *i.t*.); 16 h and 1 h before chronic constriction injury of the sciatic nerve (CCI) and then once a day for 7 days on levels of the CCL7 **(A, C)**, CCL11 **(B, D)** and CCR3 **(E, F)** proteins in the rat spinal cord **(A, B, E)** and the DRG **(C, D, F)** on the 7^th^ day post-CCI, as measured using ELISA **(A–D)** and Western blotting **(E, F)**. The data are presented as the mean fold changes relative to the control ± SEM (5–8 samples per group). Intergroup differences were analyzed using ANOVA with Bonferroni’s *post hoc* test for multiple comparisons. *P < 0.05, **P < 0.01 and ***P < 0.001 indicate a significant difference compared with the control group (naive, N rats); ^#^P < 0.05 indicates a significant difference compared with the Vehicle (V)-treated group.

Significant upregulation of the CCL7 protein was observed in the spinal cord (p = 0.0101; **Figure 7A**) and the DRG (p = 0.0038; **Figure 7C**) after nerve surgery; however, SB only prevented this change in the DRG. Moreover, a substantial increase in CCL11 levels was noticed in the DRG after sciatic nerve ligation (p = 0.0005; **Figure 7D**), and these changes were inhibited by the SB treatment. No differences were observed in the spinal cord (**Figure 7B**). After CCR3 antagonist treatment (**Figures 7E, F** **)**, the CCR3 level remained unchanged in both tested structures on day 7^th^ after nerve injury.

### Effect of Repeated i.t. SB328437 Administration on the Protein Levels of the Cellular Markers IBA-1, GFAP, CD4, CD8 and MPO in the Rat Spinal Cord and DRG 7 Days After Chronic Constriction Injury of the Sciatic Nerve in Rats

**Figure 8:**
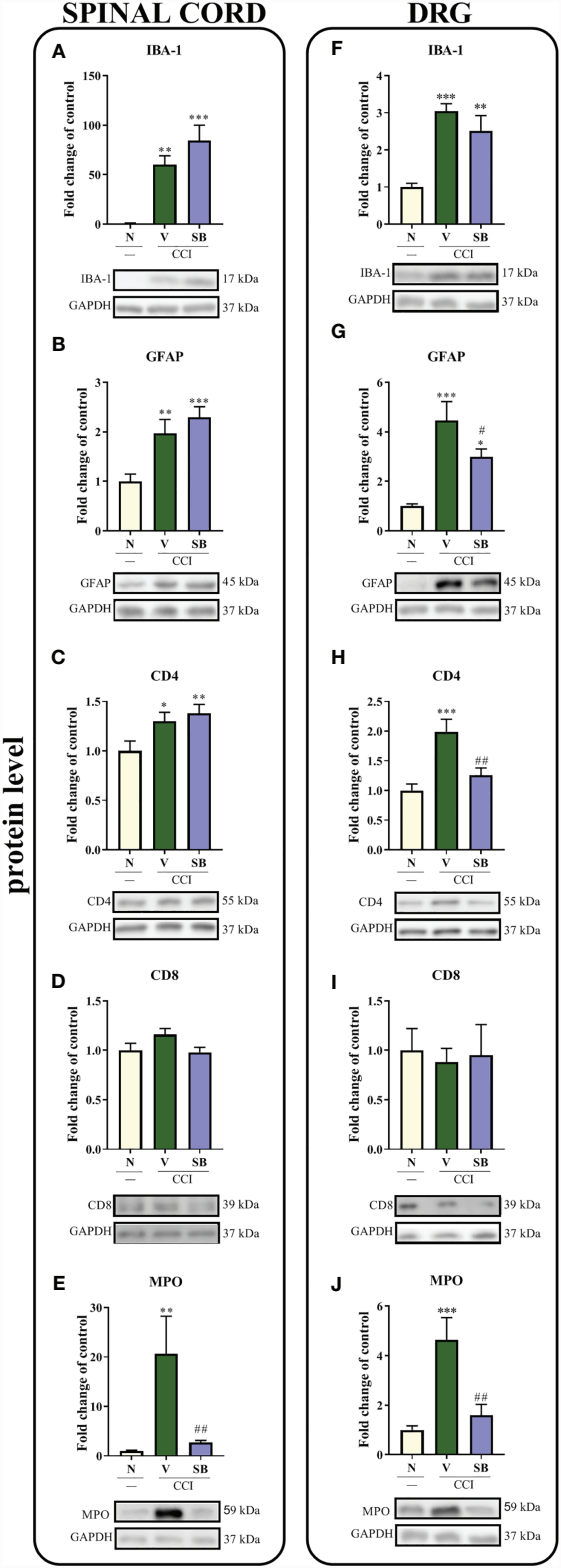
Effects of repeated administration of SB328437 (SB, 10 μg/5 μl; *i.t*.) 16 h and 1 h before chronic constriction injury of the sciatic nerve (CCI) and then once a day for 7 days on levels of the IBA-1 **(A, F)**, GFAP **(B, G)**, CD4 **(C, H)**, CD8 **(D, I)** and MPO **(E, J)** proteins in the rat spinal cord **(A–E)** and the DRG **(F–J)** on the 7^th^ day post- CCI. The data are presented as the mean fold changes relative to the control ± SEM (6–8 samples per group). Intergroup differences were analyzed using ANOVA with Bonferroni’s *post hoc* test for multiple comparisons. *P < 0.05, **P < 0.01, and ***P < 0.001 indicate significant differences compared with the control group (naive, N rats); ^#^P < 0.05 and ^##^P < 0.001 indicate significant differences compared with the Vehicle (V)-treated group.

In the spinal cord, sciatic nerve injury increased the levels of the IBA-1 (p < 0.0001; **Figure 8A**), GFAP (p = 0.0012; **Figure 8B**), CD4 (p = 0.0221; **Figure 8C**) and MPO proteins (p = 0.0035; **Figure 8E**). Importantly, repeated *i.t.* administration of a CCR3 antagonist prevented the CCI-evoked increase in MPO levels. No changes in the level of CD8 (**Figure 8D**) were obtained in either vehicle-treated or SB-treated animals compared to naive rats. Moreover, significant increases in the levels of the same markers were observed in the DRG. Interestingly, repeated injections of the CCR3 antagonist inhibited surgery-induced changes, as evidenced by the downregulation of GFAP (p = 0.0006; **Figure 8G**), CD4 (p = 0.0005; **Figure 8H**) and MPO (p = 0.0005; **Figure 8J**) protein levels, compared to vehicle-treated animals, with no significant effect on IBA-1 levels (**Figure 8F**). Similar to the spinal cord, no changes were observed in the CD8 protein level (**Figure 8I**).

### The Time Course of Changes in Levels of the MPO Protein Measured in the Spinal Cord and DRG After Chronic Constriction Injury of the Sciatic Nerve in Rats

**Figure 9:**
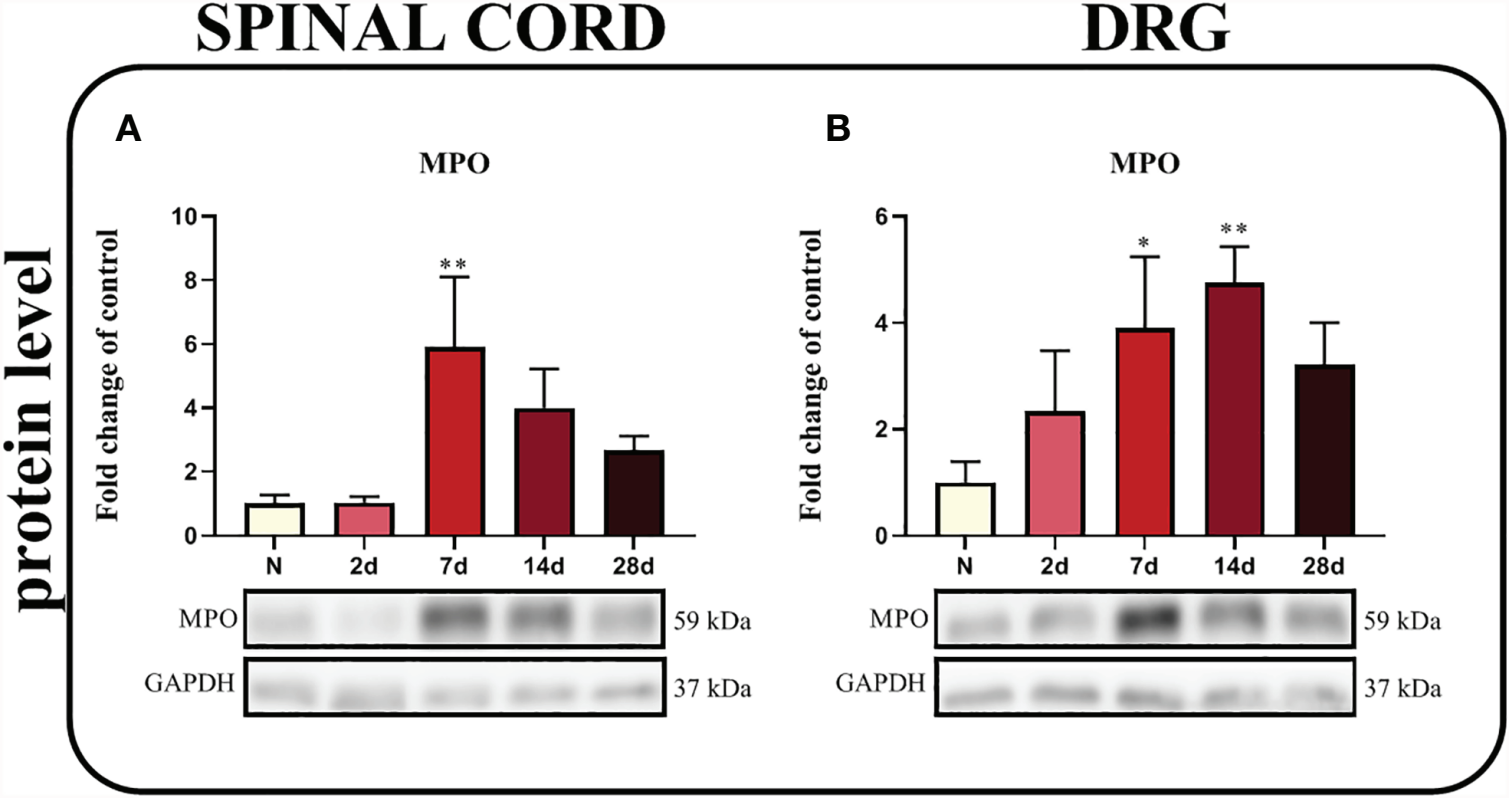
Time-dependent changes in the level of the MPO protein in the rat spinal cord **(A)** and DRG **(B)** on the 2^nd^, 7^th^, 14^th^ and 28^th^ days after chronic constriction injury of the sciatic nerve (CCI). The biochemical data are presented as the mean fold changes relative to the control ± SEM (4–5 samples per group). Intergroup differences were analyzed using ANOVA with Bonferroni’s *post hoc* test for multiple comparisons. *P < 0.05 and **P < 0.01 indicate a significant difference compared with the control group (naive, N rats).

A significant upregulation of the MPO protein was observed in the spinal cord 7 days after injury (p = 0.0330; **Figure 9A**) and in the DRG between the 7^th^ and 14^th^ days (**Figure 9B**), as determined using Bonferroni’s *post hoc* test.

### The Effect of a Single i.p. Administration of 4-Aminobenzoic Hydrazide on Morphine and Buprenorphine Analgesic Effectiveness 6 Days After Chronic Constriction Injury of the Sciatic Nerve in Mice

**Figure 10:**
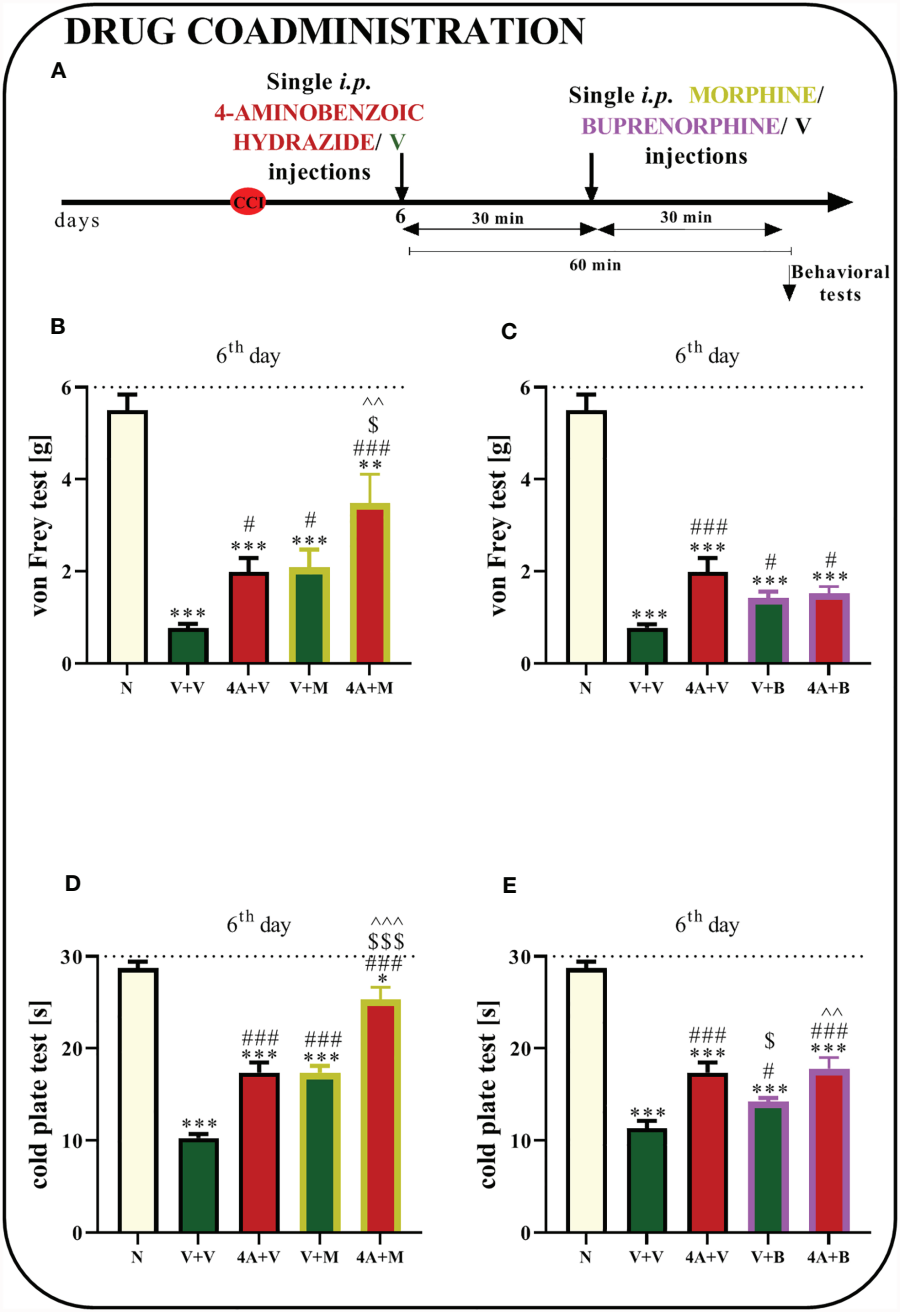
Effect of a single administration of 4-aminobenzoic hydrazide [4A; 60 mg/kg; *i.p.* **(A)**] on pain-related behaviors in mice [von Frey test - **(B, C)** and cold plate test - **(D, E)**] and the analgesic effects of morphine (M) [5 mg/kg, *i.p*.; **(B, D)**]. and buprenorphine **(B)** [5 mg/kg, *i.p.;* **(C, E)**] 1 h after vehicle or 4A injections (30 min after opioids injections) on the 6^th^ day post- chronic constriction injury of the sciatic nerve (CCI). The data are presented as the means ± SEM of 7–8 mice per group. The horizontal dotted line shows the cutoff value. Intergroup differences were analyzed using ANOVA with Bonferroni’s *post hoc* test for multiple comparisons. ^*^P < 0.05, ^**^P < 0.01, and ^***^P < 0.001 indicate a significant difference compared with the control group (naive, N mice); ^#^P < 0.05 and ^###^P < 0.001 indicate a significant difference compared with the Vehicle (V)-treated group; ^$^P < 0.05 and ^$$$^P < 0.001 indicate a significant difference compared with the 4A+V-treated group; ^^P < 0.01 ^^^P < 0.001 indicate a significant difference between the V+M- and 4A+M-treated mice and between the V+B- and 4A+B-treated mice.

Sciatic nerve surgery led to the development of mechanical and thermal hypersensitivity. Behavioral tests showed that a single 4A administration reduced mechanical and thermal hypersensitivity (**Figures 10B–E**). Moreover, the analgesic effects of morphine were stronger when morphine was coadministered with 4A, as measured using the von Frey (p = 0.0012; **Figure 10B**) and cold plate tests (p < 0.0001; **Figure 10D**). Additionally, reductions in mechanical hypersensitivity (p = 0.0010; **Figure 10C**) and thermal hypersensitivity (p < 0.0001; **Figure 10E**) were observed after buprenorphine injections. However, in the case of common administrations of buprenorphine and 4A, enhancement of opioid effectiveness was observed using only cold plate test.

## Discussion

To our knowledge, the present study is the first attempt to examine time-dependent changes in the mRNA expression of endogenous CCR3 ligands after chronic constriction injury in rats. Interestingly, our results show substantial increases in the levels of the *CCL7* and *CCL11* mRNAs in the spinal cord and/or DRG in the early and late stages of neuropathic pain. These changes were accompanied by the appearance of mechanical and thermal hypersensitivity. Additionally and most importantly, our results provide evidence that the CCR3 antagonist SB328437 reduces long-term (up to 5h) hypersensitivity to painful stimuli after both repeated and even single *i.t.* administration and enhance the analgesic properties of morphine and buprenorphine. Furthermore, repeated *i.t.* injections of a CCR3 antagonist reduce the CCI-evoked upregulation of the T cell marker CD4 and the satellite glial cell marker GFAP in the DRG, which was accompanied by the downregulation of IL-6 in the spinal cord and CCL7 and CCL11 in the DRG. Importantly, our results indicated that CCI substantially increased levels of the MPO protein in the spinal cord and DRG, changes that were diminished by a CCR3 antagonist. Moreover, an inhibitor of MPO, 4-aminobenzoic hydrazide, diminishes neuropathic pain-like behavior and beneficially influences morphine and buprenorphine effectiveness. Therefore, we postulate that CCR3-targeted therapy will be beneficial to patients since its antagonist positively influences neuroimmune interactions by modulating MPO^+^ cell activation and influx in neuropathic pain.

To our knowledge, CCR3 and its endogenous ligands (CCL5, CCL7, CCL11, CCL24, CCL26, and CCL28) have not been extensively studied in neuropathic pain conditions. In our studies, the level of the CCR3 protein remained unchanged after sciatic nerve injury, which is a common feature of many GPCRs that may be internalized after stimulation and become substituted by newly synthesized receptors (52–54). Importantly, blockade of a particular receptor can reduce pain-like behaviors; therefore, molecular studies are needed in the future to obtain a better understanding of this mechanism of action.

The pronociceptive properties of CCL5 have already been suggested; however, after sciatic nerve ligation, we did not observe its upregulation in the spinal cord or DRG, consistent with previously published results obtained on day 7 in the spinal cord (55). Similarly, *CCL24* was not upregulated, and the level of *CCL28* was undetectable. A slight increase in the expression of the *CCL26* mRNA was observed, but only on the 28^th^ day after injury; therefore, these chemokines may not be essential in the early phase of pain development. We would like to draw attention to the substantial changes in *CCL7* mRNA levels and the weaker changes in *CCL11* mRNA levels in the spinal cord and only at later time points in the DRG.

CCL7 stimulates immune cell trafficking to sites of inflammation (56), and an intrathecal injection of CCL7 evokes strong pain-like behavior, which appears after only one hour (18). Moreover, spinal nerve ligation increases the spinal expression of CCL7, and CCL7-/- mice develop symptoms of neuropathic pain to a lesser extent (45). Importantly, CCL7 neutralizing antibodies effectively attenuate neuropathic pain symptoms in mice after sciatic nerve injury (18). Ke et al. reported that neuron-derived CCL7 may promote astrocyte proliferation in response to neuropathic pain (45). Our results obtained from the 2^nd^ to 28^th^ days after nerve injury showed increased expression of the *CCL7* mRNA in both the spinal cord and DRG, consistent with previously described data obtained on the 7^th^ day. One week after CCI, we also confirmed an increase in the CCL7 protein level (55). Interestingly, the surgery-induced increase in CCL7 protein levels was inhibited by SB328437 administration in the DRG, suggesting its potentially important role in pain development in the periphery.

According to recent studies, CCL11 is also important for inflammation and nociceptive transmission (31, 43). CCL11 has a very high affinity for CCR3 and has been shown to play a crucial role in the recruitment of leukocytes and macrophages (43). Our studies revealed a significant increase in spinal expression of the *CCL11* mRNA from the 2^nd^ to 28^th^ days in the experiment and in DRG from the 14^th^ to 28^th^ days, however the spinal protein level remain unchanged at day 7. What is important the mRNA and protein level may differ. The differences between mRNA and protein levels depends on various biological and technical factors. We have to take many factors into account, such as translation efficiency, protein turnover and half-life (57, 58). Our results corresponded well with those obtained by others in mice, where gene expression microarrays performed after spinal nerve ligation revealed that *CCL11* mRNA was one of the highly upregulated chemokines on day 10 (59). Moreover, a high level of this chemokine has been observed in the cerebrospinal fluid of patients with neuropathic pain (47). Bertilimumab, a humanized monoclonal antibody against CCL11, is currently in clinical trials for treating severe allergic disorders and inflammatory bowel disease (60). Moreover, our data revealed increased levels of the CCL11 protein in the DRG 7 days after injury, and more interestingly, the use of a CCR3 antagonist prevented this increase.

Because of the significant changes in the levels of CCL7 and CCL11, the ligands of CCR3, the aforementioned receptor seems to be an interesting target for neuropathy treatment. Notably, blockade of other chemokine receptors in the CC family by their antagonists, e.g., CCR1 - J113863 (27), CCR2 - RS504393 (29), CCR4 - C021 (50), and CCR5 - maraviroc (31, 61), beneficially modulates the changes observed during neuropathy, consistent with our previous reports (27, 29, 50). Therefore, we decided to use the CCR3 antagonist SB328437 to determine whether the blockade of this receptor would also be a beneficial pain treatment. A few animal studies examining SB328437 as an experimental agent for treatment have been conducted in renal cell carcinoma (62), allergic inflammation (63) or osteoarthritis models (64); however, SB328437 treatment of neuropathic pain has not been assessed. In a chronic constriction injury model of the sciatic nerve, we observed for the first time that the aforementioned antagonist reduced pain-like behavior after repeated *i.t.* administration and, interestingly, even after a single *i.t.* injection. Not every chemokine-targeted drug is efficacious after a single injection (e.g. RS504393, a CCR2 antagonist) (55). However, SB328437 exerted analgesic effects after the administration of a single dose, similar to other substances that act *via* chemokine receptors, e.g., J113863 - a CCR1 antagonist or cenicriviroc - a CCR2/CCR5 antagonist (27, 55). Our results provide evidence that CCR3 is an interesting target in pain research. Therefore, we also investigated whether and how the blockade of CCR3 by its antagonist affects the cells known to be involved in neuropathic pain pathogenesis.

Microglia are intimately involved in the development and maintenance of neuropathy and pain-like behaviors (65, 66). These cells may become activated, proliferate and release nociceptive factors following nerve injury (67, 68). Here, in our studies, we measured the spinal level of IBA-1, a marker of microglial/macrophages activation and proliferation. The obtained data indicated a substantial upregulation of IBA-1 7 days after nerve injury, consistent with previous data (27, 31, 50). Similar observations were recorded in the DRG, where the expression of the macrophage marker was increased, consistent with previous papers (27, 50). Surprisingly, in contrast to the antagonists of CCR1 - J113863 (27), CCR2 - RS504393 (29), CCR4 - C021 (50), and CCR5 (31) - maraviroc, the CCR3 antagonist SB328437 did not alter CCI-upregulated IBA-1 marker in the spinal cord or DRG; however, pain-like behavior symptoms were diminished.

The second population of nonneuronal cells involved in modulation of nociceptive transmission in the spinal cord are astrocytes. GFAP^+^ cells in the central nervous system are well known to be activated in the spinal cord after peripheral nerve injury (27, 31, 69, 70), and our results are consistent with these data. Moreover, SB328437 did not prevent spinal GFAP upregulation after nerve injury; however, upon injury, activated astrocytes may exert not only harmful but also protective functions, such as the promotion of neuronal survival, neurogenesis or remyelination (71). In the DRG, the effect of satellite glial cells (SGCs) on the production of pronociceptive factors after nerve injury has also already been confirmed (72). These cells, among others, may be responsible for the development and persistence of neuropathic pain. A commonly reported change in these cells occurring during neuropathy is proliferation and activation (73, 74). Furthermore, activated SGCs release pronociceptive cytokines that act on neurons, leading to an increase in excitability (75). Additionally, nerve injury may lead to a disruption of K^+^ homeostasis regulated by Kir4.1 channels, which are expressed on SGCs and consequently may intensify pain (72). SB328437 decreased the CCI-evoked upregulation of satellite glial cell marker GFAP in the DRG, which may be associated with maintaining perineuronal environment homeostasis and, as a possible consequence, diminished pain-like behaviors.

Recently, evidence has emphasized that neutrophils are also important participants in neuropathic pain pathogenesis (76). Neutrophil infiltration into the DRG was observed after peripheral nerve injury (27, 77). Additionally, these cells invade the spinal cord under conditions of neuropathy (78). One of the most abundant proteins in neutrophil granules is MPO. The substantial upregulation of this enzyme was observed in our studies both in the spinal cord and DRG after CCI, especially on days 7 and/or 14 after CCI. These changes may be associated with increased infiltration and activation of neutrophils. MPO also activates nociceptive neurons to subsequently induce pain (79). Moreover, MPO is known to be involved in the production of hypochlorous acid (HOCl), which may participate in tissue injury associated with inflammation (80). Additionally, strong activation of this enzyme was observed in the sciatic nerve of rats after injury (81) and following treatment with chemotherapeutics such as paclitaxel (82, 83) and oxaliplatin (84), which were accompanied by pain-like behaviors. Interestingly, SB328437 reduced the CCI-induced increase in MPO levels, which might be associated with decreased levels of infiltrating neutrophils, to confirm that, further studies are required. Another possible explanation for the decreased MPO levels is the phagocytosis of neutrophils or neutrophil extracellular traps (NETs) by activated macrophages (85–87), and increased IBA-1 levels may be a good indicator of this process since these proteins are involved in membrane ruffling and phagocytosis (88). Moreover, our studies are the first to show that inhibition of MPO activity by 4-aminobenzoic hydrazide reduces mechanical and thermal hypersensitivity, confirming the important role of neutrophils in neuropathy.

Notably, the release of proinflammatory factors by neutrophils may lead to T cell activation (89). The effect of CD4^+^ T cells on neuropathy is well known (14, 90). Evidence suggests that these lymphocytes may invade the sciatic nerves, DRG and spinal cord after peripheral nerve injury (10, 91). Moreover, athymic nude rats (rnu−/−, lacking mature T cells) exhibit reduced mechanical and thermal hypersensitivity compared to heterozygotes after CCI (10). In our experimental model, we observed an upregulation of CD4 in the DRG after sciatic nerve injury, and the CCR3 antagonist prevented this change. Therefore, in our opinion reducing CD4^+^ T cells activation and infiltration, in combination with changes observed in other cells, may lead to the attenuation of peripherally derived pain. No changes were observed for CD8 expression, similar to other studies (27).

Moreover, the abovementioned nonneuronal cells release factors with pro- and antinociceptive properties after nerve injury. As described above, SB328437 prevented increases in CCL7 and CCL11 levels. However, more factors were influenced by the CCR3 antagonist. In our research, we observed the upregulation of the proinflammatory cytokines IL‐6 and IL‐18 in the spinal cord and the DRG after nerve injury, consistent with other studies showing their involvement in neuropathic pain (27, 29). Based on the results of our study, the CCR3 antagonist altered the level of spinal IL-6 but not IL-18 after sciatic nerve injury. In our opinion, a reduction in the level of IL-6 contributed to pain relief since neutralization of IL‐6 leads to the attenuation of hypersensitivity symptoms (92). Furthermore, IL-6-/- mice exhibit less mechanical hypersensitivity after nerve injury (93). Recently, an increasing number of studies have highlighted an important role for antinociceptive interleukins, such as IL-10 (94, 95), IL-1RA (96) and IL-18BP (97), in pain processes. The analgesic properties of maraviroc [a CCR5 antagonist (98)] and J113863 [a CCR1 antagonist (27)] increase the levels of IL-1RA, IL-18BP and/or IL-10. However, our results did not show an effect of SB328437 on the levels of antinociceptive factors after nerve injury. Therefore, we postulate that pain relief induced by targeting CCR3 may be more related to the effect on pronociceptive factors.

Opioids are drugs that are used to treat severe types of pain; however, their effectiveness in neuropathy is weaker (9). Morphine was suggested to have lower analgesic efficacy in neuropathic pain because of the reduced number of presynaptic opioid receptors caused by the degeneration of primary afferent neurons after nerve damage (99). Our study indicates that SB328437 improves the analgesic properties of morphine, as measured using the von Frey and cold plate tests, and buprenorphine to a lesser extent, where the CCR3 antagonist influences only mechanical hypersensitivity. The mechanism by which chemokine receptor antagonists modulate opioid efficacy remains unclear, but several hypotheses have been proposed. One may be the heterologous desensitization of chemokines and opioid receptors. The finding of MOR-CCR5 heterodimerization provides an interesting level of possible complexity in the negative cross-talk between chemokine-opioid receptors, during which the inhibition of one dimer partner causes the activation of the other (100–102). Moreover, literature data give evidence that CCR3 and opioid receptors are present on the same cell types, including those important for nociceptive transmission like microglia (34, 36, 103), astroglia (36, 104, 105) and neutrophils (37, 106, 107) that is why we think that heterodimerization may be possible. The second potential mechanism of different opioid effectiveness in neuropathy may be attributed to the effect of chemokine receptor antagonists on the activation of immune and glial cells. It has been suggested that uncontrolled activation of glia leads to enhanced activity of opioid systems or opioid-specific signaling (108–110). Interestingly, studies showed that astroglial and microglial activation is enhanced after opioid treatment, which also has consequences for opioid pharmacodynamics (111). Furthermore, it has been suggested that the suppression of immune and glial cell activation induces the inhibition of nociceptive cytokine synthesis, which in turn can improve morphine efficacy in treating neuropathic pain (109, 112, 113). We were surprised to find out that the intensification of opioid effects was not accompanied by the decrease in microglial and astroglial activity, since these cells are often involved in this mechanism (114). Our previously published papers have shown that the blockade of several chemokine receptors diminishes microglial activation: CCR1 – J113863 (27), CCR2 – RS504393 (29), CCR4 – C021 (50), CCR5 – maraviroc (31), and CXCR3 – (±)-NBI-74330 (32), and enhances morphine/buprenorphine analgesia through this mechanism. In contrast, SB328437 acts probably by other mechanisms, since it does not affect activation of IBA-1-positive cells but still enhance opioid effectiveness. Since we did not observed changes in markers of IBA-1 and GFAP in the spinal cord, we decided to assess whether this potentiation of opioid effects was achieved by modulating neutrophils and, more specifically, by inhibiting MPO, since no evidence is available showing those possible mechanisms. Interestingly, our research was the first to show that 4-aminobenzoic hydrazide alters morphine effectiveness, as measured by von Frey and cold plate test and buprenorphine, however only through the impact on thermal hypersensitivity, indicating that a reduction in MPO activity contributes to the intensification of its analgesic effects. These results provide evidence that SB328437, through its effect on MPO, may participate in the potentiation of opioid analgesia; however, more studies are needed to explore the full mechanism.

## Conclusions

In summary, we are the first to identify CCR3 as a promising target for neuropathic pain therapy. Our study provides evidence that SB328437 may reduce mechanical and thermal hypersensitivity induced by nerve injury after repeated and even single administration when symptoms of neuropathic pain are fully established. Moreover, blockade of CCR3 enhances morphine and buprenorphine effectiveness. The observed beneficial effects are related to the fact that SB328437 prevents nerve injury-induced upregulation of neutrophils, satellite cells and CD4+ T cells markers while upregulating IL-6, CCL7 and CCL11. Our results support the hypothesis that the pharmacological modulation of neuroimmunological interactions by CCR3 may represent a new strategy for effective pharmacotherapy in pain treatment. Interestingly, we have shown that treatments targeting neutrophils that inhibit MPO activity may also prevent pain-like behaviors and enhance opioid efficacy. Because of these promising results, we propose that treatments targeting chemokine receptors will be explored more extensively in the future and will expand the range of commercially used drugs.

## Data Availability Statement

The original contributions presented in the study are included in the article/supplementary material. Further inquiries can be directed to the corresponding author.

## Ethics Statement

Experiments were carried out according to the recommendations and standards of the International Association for the Study of Pain (IASP) and the National Institutes of Health (NIH) Guide for the Care and Use of Laboratory Animals and were approved by the Ethical Committee of the Maj Institute of Pharmacology of the Polish Academy of Sciences (LKE: 116/2021; 213/2021; 1277/2015). According to the 3R policy, the number of animals was reduced to the necessary minimum.

## Author Contributions

KP, AC, KC, ER, WM and JM substantially contributed to the conception and design of the study and to the analysis and interpretation of data.

## Funding

This work was supported by the National Science Center, Poland, grant OPUS 11 2016/21/B/NZ4/00128 and statutory funds from the Maj Institute of Pharmacology Polish Academy of Sciences.

## Conflict of Interest

The authors declare that the research was conducted in the absence of any commercial or financial relationships than could be construed as a potential conflict of interest.

## Publisher’s Note

All claims expressed in this article are solely those of the authors and do not necessarily represent those of their affiliated organizations, or those of the publisher, the editors and the reviewers. Any product that may be evaluated in this article, or claim that may be made by its manufacturer, is not guaranteed or endorsed by the publisher.
